# Bulge Region as a Putative Hair Follicle Stem Cells Niche: A Brief Review

**Published:** 2017-09

**Authors:** Sanaz JOULAI VEIJOUYE, Abazar YARI, Fatemeh HEIDARI, Nayereh SAJEDI, Fatemeh GHOROGHI MOGHANI, Maliheh NOBAKHT

**Affiliations:** 1. Physiology Research Center, Iran University of Medical Sciences, Tehran, Iran; 2. Dept. of Biology, University Campus 2, University of Guilan, Rasht, Iran; 3. Dept. of Anatomy, School of Medicine, Alborz University of Medical Sciences, Karaj, Iran; 4. Dept. of Anatomy, School of Medicine, Qom University of Medical Sciences, Qom, Iran; 5. Dept. of Anatomy, School of Medicine, Isfahan University of Medical Sciences, Isfahan, Iran; 6. Dept. of Anatomy, School of Medicine, Tehran University of Medical Sciences, Tehran, Iran; 7. Anti-Microbial Resistance Research Center, Iran University of Medical Sciences, Tehran, Iran

**Keywords:** Bulge, Hair follicle, Stem cell, Regeneration, Pluripotent, Self-renewal

## Abstract

**Background:**

Hair follicle stem cells exist in different sites. Most of the hair follicle stem cells are reside in niche called bulge. Bulge region is located between the opening of sebaceous gland and the attachment site of the arrector pili muscle.

**Methods:**

Data were collected using databases and resources of PubMed, Web of Science, Science Direct, Scopus, MEDLINE and their references from the earliest available published to identify English observational studies on hair follicle bulge region.

**Results:**

Bulge stem cells are pluripotent with high proliferative capacity. Specific markers allow the bulge cells to be isolated from mouse or human hair follicle. Stem cells isolated from bulge region are label retaining and slow cycling hence these cells are defined as label-retaining cells. Bulge cell populations, due to their plasticity nature are able to differentiate into distinct linage and could contribute in tissue regeneration.

**Conclusion:**

The current review discuss about bulge stem cells characteristics and biology including their cycle, location, plasticity, specific markers and regenerative nature. Also the differences between mouse and human hair follicles are investigated.

## Introduction

In recent years, the identification and characterization of adult stem cells was one of the biological and biomedical research interests (1). Adult stem cells with slow-cycling nature are capable to differentiate into all cell types, and they are self-renewal in order to refill the stem cell pool. These characteristics make them responsible to regenerate many tissues (2,3). Adult stem cells can be found in various tissues, including hematopoietic system, skeletal muscle, nervous system, liver and epidermis (3) as well as integument appendages such as feathers, teeth and hair follicles. Hair follicles stem cells are reserved in niche called bulge (1). Bulge is located between the opening of sebaceous gland and the attachment site of the arrector pili muscle (4). Bulge stem cells are multipotent and have high proliferative potential (5). These cells generate all epithelial lineages of the skin, including keratinocytes, sebocytes and hair (4).

Hair follicles reconstitute during the cycle that initiate with growing phase (anagen), followed by regression phase (catagen) and finally resting phase (telogen) (6).

Since in recent years, adult stem cell therapy plays an important role in clinical application, thus more investigating about hair follicle stem cells may lead to treatments of injuries and diseases, therefor the aim of this study is to discuss about bulge stem cells characteristics and biology.

## Methods

Databases and resources of PubMed, Web of Science, Science Direct, Scopus, and MEDLIN from the earliest available online indexing year through 2016 were searched. The search keywords included bulge, hair follicle, stem cell, marker, regeneration, differentiation, pluripotent, and self-renewal. The reference lists of articles were read to obtain additional information. Only the English publications were studied. Lately published cohort and case-control studies on the interesting topic are cited. First, the titles and abstracts of all retrieved studies were screened, then the full texts of potentially eligible studies were reviewed before definitive inclusion and relevant data were extracted from studies.

### Hair Follicle Anatomy

Hair as one of the skin appendages grows from hair follicle (7). Hair follicle is composed of 3 regions: the lower part (bulb and suprabulb) that begins from the follicle base and continuous to the insertion of the arrector pili muscle, the middle part (isthmus) is a short section that begins from insertion of the arrector pili muscle and continuous to the entrance of the sebaceous gland duct and the upper part (infundibulum) that begins from the entrance of the sebaceous gland duct and continuous to the follicular orifice. The bulbous part in the lower hair follicle is hair bulb which includes of the dermal papilla and matrix. Suprabulb segment is composed of hair shaft, root sheath; glassy layer and fibrous root sheath extends from bulb to the isthmus. Hair shaft is made up of 3 layers: cuticle, cortex and medulla (8). Hair root is consisting of 2 layers: outer root sheath (ORS) and inner root sheath (IRS). IRS is consisting of 3 layers from out to in: Cuticle, Huxley and Henle (9). The IRS extends from matrix cells to entrance of the sebaceous gland duct. Between arrector pili muscle and sebaceous gland duct IRS make bulge region which is the reservoir of the stem cells (2). The outermost layer of follicles is the fibrous root sheath that covers the entire follicle (8).

### Hair Follicle Cycle

Hair growth rather than occurring continually is an alternating cycles of growth and quiescence (10). For generating new hair, hair follicles undergo cyclical changes consist of anagen (growth), catagen (regression), telogen (rest) and exogen (11).

In each growth cycle, for generating transiently amplified cells, stem cells resident in the niche proliferate, subsequently a differentiation process occurs and the new hair shaft is built (2). Indeed, hair bulge stem cells are responsible for generating the follicle in hair cycle (12).

Follicles generate hair shaft during anagen stage, subsequently within catagen and telogen follicles reset to be able to receive signals in order to onset the next cycle (13). BMP and WNT signaling supposed to play an important role in onset of anagen (14).

Matrix keratinocytes located in the hair follicle bulb, rapidly proliferate and form the hair during anagen.

The duration of anagen is variable in hairs of different sites (5). Accordingly the hair shaft size depends on the anagen period (14). For instant anagen duration in human scalp follicles with 5 mm long hair lasts for many years while in mouse pelage follicles with the length of 1 mm hair it is only 2–4 weeks (5, 14).

By the end of anagen, epithelial cells below the bulge stop proliferation and undergo apoptosis; in the meanwhile the dermal papilla mesenchyme cells survive and after entering into catagen phase form the club hair (7). During telogen these cells move to undermost bulge region, and generate secondary germ (14). Right after telogen, by starting a new anagen phase, while the new hair grows the club hair is shed during exogen (5).

LRC population localized at bulge area, it was though that, the hair follicle bulb area harbors stem cell and during anagen phase, the secondary germ ambulate to the follicle bulb in order to produce new hair, and in return during catagen, secondary germ move upward with dermal papilla (5) (15).

### Bulge Stem Cells

Hair follicle stem cells (HFSCs) are major source of pluripotent adult stem cells (16). These cells, like other adult stem cells are slow-cycling cells with the proliferative capacity and ability to generate various tissues (17) HFSCs are reserved in niche called bulge (13). Bulge is located between the opening of sebaceous gland and the attachment site of the arrector pili muscle (4). Unna believed that growth of club hair is from bulge region; hence he gave the term “Haarbett” (hair bed) to this region. Stöhr named it “Wulst” (bulge or convexity) (5).

To identify adult stem cells, the main method is using their slow cycling nature. Generally all the cells of a tissue are labeled with a DNA precursor, including bromodeoxyuridine (BrdU) or tritiated thymidine (3H-T). In this process, only the slow cycling cells retain their labels while rapidly dividing cells lose most of their labels (17). In this way, the labeled slow cycling cells defined as label-retaining cells (LRCs) (6). Label-retaining cells (LRC) with slow cycling nature are reserved in the bulge region of hair follicles (15). Morris et al. declared that bulge cells are label-retaining for 14 months which is equal to the whole lifespan of mice (18). After it became clear that bulge is reservoir of LRC, many other researchers try to study properties of bulge LRC (1).

### Bulge Stem Cell Markers

To identify and isolate stem cells, obtaining specific stem cell marker is essential. Likewis e, detecting bulge specific marker helps to identify the existent stem cells. Hence researchers utilized many experiments for the purification of bulge stem cells (19–21).

Keratin 15 (K15) is an intracellular intermediate filament protein; this marker has high expression in the bulge region. Indeed the LRC reservoir is K15 positive (14).

It is notable that K15 has low level expression in the lower follicle. Thus K15 is not limited to bulge cells and it is not a very specific marker of this region (5, 22). However, K15 promoter in adult transgenic mice carrying LacZ gene, express LacZ confined to hair follicle bulge (6). Morris et al. used K15 promoter to stimulate expression or enhanced green fluorescent protein (EGFP) or LacZ gene in transgenic mice. These expressions are restricted to the LRC of bulge area. These processes allow isolating bulge cells using FACS-based sorting (23).

For the first time Lyle et al. found that human bulge cells are K15 positive (20). Ohyama et al. used K15 marker to isolate stem cells of human hair follicle. Using microarray analysis investigated many expressed genes in human bulge cells (22).

CD34 is a surface protein, which is recognize as a specific marker of mouse hair follicle bulge stem cells (24) but not obvious in human bulge area (25,26). Bulge LRC are CD34 positive, Trempus et al. declared CD34 as mouse bulge marker for the first time. Using CD34 antibodies, bulge cells were collected by FACS (21,24). In human bone marrow, CD34 is found as hematopoietic stem cell marker (6).

Ohyama et al. discovered that human bulge LRC expressed CD34 in low level (22). Consequently CD34 is identified as an inappropriate marker for human bulge cells, but a suitable mouse bulge marker. Thus CD34 plays an important role in bulge cells studies (6). Nestin is an intermediate filament. Several groups found that Nestin as a marker of neural stem cells, expresses in bulge cells population (27–29).

Nobakht et al. limited their experiments on bulge area of rat vibrissa and found that stem cell marker including Nestin and CD34 were expressed in rat vibrissa bulge cells (28). On the other hand, the expression of Krt19, Lgr5 and the transcriptional factors Gli1, Sox9, Hopx, Nfatc1, Tcf3 and Lhx2 could be markers to identify mouse bulge stem cells (2).

**Fig. 1.**
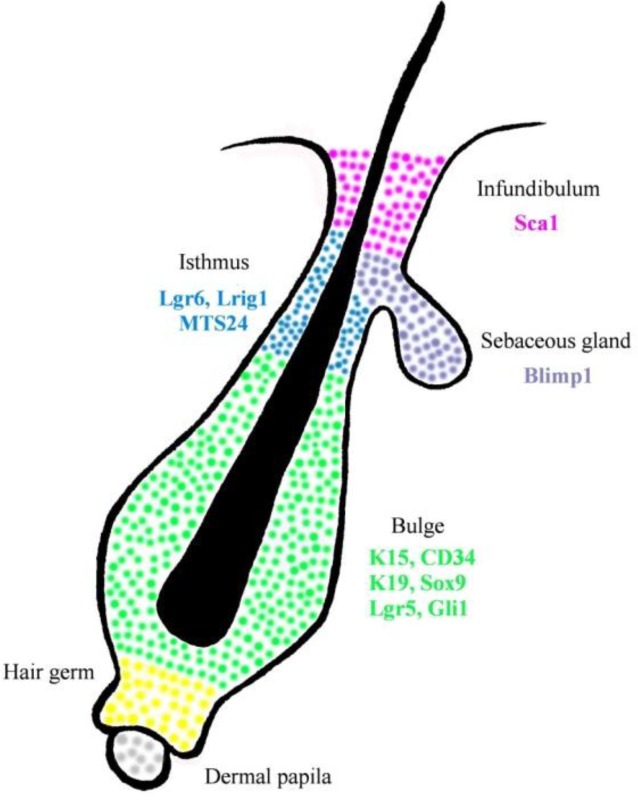
Hair follicle stem cells distribution. Different parts of hair follicle could be identified by specific markers. The bulge stem cells (green) are K15+, CD34+, K19+, Sox9+, Lgr5+ and Gli1+. Sebaceous gland (purple) are Blimp1+, Isthmus cells (blue) are MTS24+, Lgr6+ and Lrig1+. Sca-1+ cells (pink) are found in infundibulum.

Other parts of hair follicle could be identified by other specific markers (Fig. 1). Isthmus cells are MTS24+, Gli1, Lgr6+ and Lrig1+ (30). Isthmus cells are CD34− and Krt15− (2). In compare, infundibulum cells are Sca-1+ (12) junctional zone contain Lrig1+ cells (12,30) and Blimp1+ cells found in sebaceous gland (1) (Table 1).

**Table 1:** Hair follicle stem cells markers. This table contains some of hair follicle putative markers

**Marker**	**Location**	**Reference**
**CD34+**	Bulge	(24)
**K15+**	Bulge	(23)
**K15 promoter**	Bulge	(23)
**Nestin**	Bulge	(27)
**CD200+**	Bulge	(33,22)
**K19+**	Bulge	(2)
**Lgr5+**	Bulge	(2)
**Gli1+**	Bulge	(2)
**Sox9+**	Bulge	(2)
**Hopx+**	Bulge	(2)
**Nfatc1+**	Bulge	(2)
**Tcf3+**	Bulge	(2)
**Lhx2+**	Bulge	(2)
**Lgr6+**	SG (isthmus)	(2)
**Lgr1+**	Isthmus	(12)
**Sca1+**	Infundibulum	(12)
**Blimp1+**	SG	(1)

The bulge area is also reservoir of melanocyte stem cells (MSCs). Melanocytes proliferate to re-populate the melanocytes which raise melanin in order to accomplish hair pigmentation (31).

### Human In Compare With Mouse Hair Follicle

Major researches for studying hair follicle stem cells are performed on mouse models. Significant differences exist between microanatomy of mouse and human hair follicle stem cells. Hence results from mouse models are necessary to be confirmed in human organism (14). Since the morphology of hair follicle bulge is difference in human and mouse, better understanding of human bulge area is essential.

In mouse pelage hair follicle, bulge is specified as a distinct swelling section of outer root sheath. Despite human bulge is also well identified in embryonic hair follicle, it is found as a petty distend in adult hair follicle (6).

Human scalp follicles are 5 mm long, while the mouse pelage follicles are 1 mm in length (14). Another difference that can be noted is that the hair cycle has longer period in human hair follicle than in mouse. Each cycle of human hair follicle lasts about a decade, though it last some weeks in mouse follicles (25). Studying of rat whisker (vibrissa) follicles showed that, these follicles have larger size and unusual structure in compare with pelage follicles. As it is clear, obvious differences could be found between human and mouse follicle stem cells characteristics (14). On the other hand, LRC are resident in both mouse and human bulge structure (20,22).

Ohayama et al. found many identical and different genes that express in human and mouse bulge cells (14), and identified that among these markers CD200 express in human bulge in high level but CD34 has low level expression (6).

CD200, a membrane glycoprotein is a subset of immunoglobulin superfamily (32). Using microarray analysis, CD200 was identified as an authentic human bulge cells marker (4), but not mouse bulge stem cells (12). In contrast to human bulge cells K15 (23) and CD34 (21) are specific markers of mouse bulge stem cells which are not identified as human bulge cells (14) (12) (Table 1).

### Gene Regulation

Gene expressions control the noncyclic nature and quiescent of hair follicle stem cells, defining gene expression that distinct bulge and non-bulge cell populations provide intuitions of the maintenance of the stem cell (14). During adult life when the hair follicle is in the rest phase, many different genes are expressed in the bulge region. It becomes complex according to the overlap of markers between different parts of hair follicle niche and the distribution of niche markers during active hair growth (2).

A combination of bulge cell isolation and multiple gene expression microarray analysis allows distinguishing bulge stem cells form other differentiated cells. Mouse bulge cells molecular signatures by using different isolation methods were obtained (6).

Bulge cells microarray results declared that genes involved in morphogenesis, organogenesis and development are up regulated, in contrast gene related to cycling and proliferation were down regulated, these results were compatible with quiescent property of bugle stem cells (33). These finding reveal how bulge stem cells of mouse adult hair follicle are quiescent and remain in un-differentiated state. In human and mouse bulge cells, WIF1 and DKK3 as inhibitors of WNT and BMP signaling pathways were down regulated (6).

Immunohistological staining declared that K19 and β1-integrin were highly expressed in bulge area (3). CD34 is exclusively express in the bulge, while K15, K19, Lgr5 and Sox9 display in bulge and hair germ. Similarly the expression of Gli1 (the transcription factor) is found in the upper bulge and the hair germ. Integrin 6 is display in bulge cells in different amount according to the vicinity of these cells to the basement membrane (2).

On the other side, human bulge cells surface markers were identified by microarray analyses, such as CD200 and CD59 which are up regulated in bulge and CD24, CD34, CD71 and CD147 are down regulated. Among these markers CD200 is the best human bulge cells marker. In addition, frizzled homolog 1 (FZD1), was up regulated in human bulge cells.

Interestingly, some human bulge up regulated genes including DIO2 and ANGPTL2 were not expressed in mouse bulge cells. The mouse bulge cell marker CD34 were expressed in human bulge in low level (6).

### Hair Follicle Signaling Pathways

Hair follicle development and physiology are under control of signaling cascade. Signaling pathways regulate quiescent, differentiation and proliferation of hair follicle stem cells, including BMP, Wnt, Shh, Notch, FGF and TNF (1–4). For hair follicle development and cycling, Wnt and BMP signaling pathways are required (6). Wnt/β-catenin signaling pathway plays an important role in morphogenesis initiation and onset of hair cycling (anagen) (35). Indeed, cell fate regulation is under control of Wnt/β-catenin signaling. Wnt as secreted glycoprotein, prompt a cascade by binding to Frizzed receptors which results in shifting APC/Axin/GSK-3β complex into GSK-3β. When Wnt signaling is absent, β-catenin is phosphorylated, hence could be degraded by APC/Axin/GSK-3β complex. When Wnt is available, Dishevelled is phosphorylated and as result the complex converts to GSK-3β. All these events cause β-catenin stabiles which leads to activation of transcription. Connection between Wnt signaling and TCF3 transcription factor is require for hair follicle stem cells maintenance. In the case of Wnt suppression, stem cells differentiate into sebocytes and keratinocytes. Wnt signaling directs mouse hair follicle bulge stem cells to preserve the stem cell circumstance, also the permission to fulfill a differentiation pathway is under control of Wnt signaling pathway (12). Interestingly Wnt signaling is also needed for matrix proliferation (2).

However, Wnt and some other signals induce stem cell proliferation while BMP signals suppress cell activation. Major BMP signaling role is regulating terminal differentiation of hair shaft and IRS. BMP and FGF18 signals secreted from dermal papilla, the internal K6+ bulge area and bulge stem cells, these signals make bulge stem cells retain their slow-cycling nature. When Wnt signal activates, BMP signal suppresses and results in onset of hair germ proliferation (2).

Another signaling pathway that control hair follicle stem cell function is sonic hedgehog (Shh) signaling. Shh is a second important signaling pathway that controls hair follicle bulge stem cells (12). Shh/Gli signaling pathway contributes in generating a growing hair follicle by inducing hair progenitor proliferation (35). Shh is a ligand which binds to Patched (Ptc), afterward Ptc binds to Smoothened (Smo) and cause it’s inhibition. At downstream of Smo, the Shh signaling assembles. After binding shh to Ptc, Smo is phosphorylated. Then Ci protein released into nucleus and stimulates Shh target genes expression. Within embryonic development, Shh suppression cause the follicle remains at bud stage (12). Regulating of mature hair follicle differentiation is under Notch signaling control (36).

### Bulge Stem Cell Multipotency

Stem cells due to their mulipotent nature are able to reorganize different lineages in the specific tissue. To study multipotnecy of bulge stem cells, various researches have been performed (6). Bulge stem cells proliferate at the beginning of anagen (5).

Taylor trace bulge stem cells of pelage follicle using double labeling, in lower follicle, faint labeling was detected in some cells. These results indicating that, these cells had originated from bulge region (37). Elsewhere, Tumber et al. used GFP labeling to demonstrate that lower epithelial cells had originated from bulge (19). None of these experiments were able to prove that matrix keratinocytes were originated from bulge, and both were unable to certainly mark bulge cells and their progeny (5).

To study bulge cells multipotency, Oshima et al. focused on transplantation. They worked on ROSA26 (transgenic mice) that express lacZ consistently. They dissected the bulge area from ROSA26 vibrissa follicles. Afterward, labeled bulge were transplanted into the vibrissae of non-ROSA26 mice. After downward migration of bulge cells through vibrissa follicle, expression of lacZ in all epithelial cell layers of some follicles were observed (38).

Moriis et al. to study about bulge cells mulipotency used K15 promoter targeted with CrePR1. K15-CrePR1 transgenic mice were crossed with R26R (reporter mice) which could express lacZ under control of ROSA26 promoter. lacZ expression was observed in bulge cells and all their progeny. This experiment made it clear that bulge progeny could generate all epithelial cell layers located in the lower hair follicle (23).

Interestingly, after culturing isolated bulge cells *in vitro*, repopulation of hair follicle, epidermis, sebaceous gland and bulge cells was observed, these results support the multipotency nature of bulge stem cells (24,39).

### Bulge Stem Cells Contribution in Wound Healing

In addition to the production of a new hair follicle, bulge stem cells are able to regenerate and contribute in tissue regeneration. During an injury, bulge cells migrate into damaged epidermis in order to repair the wound (6, 30). Besides, hair follicle keratinocytes contribute in wounds repopulation by emerging from the follicle. Ito et al, labeled bulge cells using K15CrePR;R26R transgenic mouse, ulceration with trephine lead the bulge cells progeny to migrate into the healing epidermis. The results showed that the origin of the formed epidermis were 25% from bulge cells (5,30).

### Bulge Stem Cell Plasticity

Some cell populations, due to their plasticity nature are able to differentiate into other linage including neural cells (6).

Amoh et al. isolated Nestin positive cells from mouse hair follicle bulge, then cultured these cells *in vitro* and subjected to classIII beta-tubulin +ve neuronal type cells, then cells were transplanted into nud mice, the result showed that these cells were able to differentiate into several cell lineages including neurons, Schwann cells, glia, keratinocytes, melanocytes and smooth muscle cells. In injured sciatic nerve, implanted Nestin positive cells were able to differentiate into Schwann cells and repair the severed nerve. Thus bulge cells could contribute in tissue repairing by their plasticity nature (27,40).

Nobakht et al experiment on rat vibrissa bulge cells showed that these cells were able to differentiate into neural and glia lineages (28). Drewa et al. studied on bulge stem cells of rat vibrissa and found that these cells could be used for in vitro restoration of urinary bladder wall grafts (41).

Most of studies were experimented on mouse but not humans (6). However some other findings showed that human follicle cells also demonstrated the plasticity feature (42).

According to these findings, the hair follicle could be considered as a potential source of stem cells in tissue engineering (5).

## Conclusion

The hair follicle structure is a remarkable model for studying preserved stem cells within niches. For this purpose, numerous studies have been developed on biology of bulge stem cells. The results demonstrated that label-retaining cells with mulitipotent potential and quiescence feature are reserved in hair follicle bulge region. For isolating bulge cells, specific markers of mouse and human bulge cells such as K15 promoter activity, CD34 and CD200 are needed. Since significant differences are exist between rodent and human hair follicle stem cells, obtaining results from mouse should be study on human directly. Bulge stem cells are able to differentiate into different lineages. In bulge cells differentiation process, signals including Wnt and Shh play an important role.

All of these studies about bulge stem cells may lead to finding novel techniques for utilizing stem cells in clinical application.

## Ethical considerations

Ethical issues (Including plagiarism, informed consent, misconduct, data fabrication and/or falsification, double publication and/or submission, redundancy, etc.) have been completely observed by the authors.
